# Supplementary material for: Pathways to school success: Self-regulation and executive function, preschool attendance and early academic achievement of Aboriginal and non-Aboriginal children in Australia’s Northern Territory
Source: PLoS One. 2021 Nov 11;16(11):e0259857. doi: 10.1371/journal.pone.0259857 (PMC8584680; doi:10.1371/journal.pone.0259857)
Supplement: S1 File — (DOCX) [file pone.0259857.s001.docx]

**S1 File**

## S1 Table. Sub-domain items of AEDC included in developing the latent construct for the indicator of self-regulation and executive function

| **Sub-domain: Overall Social Competence (8 items)** |
| --- |
| 13. How would you rate this child’s overall social/emotional development? |
| 14. How would you rate this child’s ability to get along with peers? |
| 15. Would you say that this child plays and works cooperatively with other children at the level appropriate for his/her age? |
| 16. Would you say that this child is able to play with various children? |
| 29. Would you say that this child is able to solve day-to-day problems by him/herself? |
| 30. Would you say that this child is able to follow one-step instructions? |
| 31. Would you say that this child is able to follow class routines without reminders? |
| 32. Would you say that this child is able to adjust to changes in routines? |
| **Sub-domain: Responsibility and respect (8 items)** |
| 17. Would you say that this child follows rules and instructions? |
| 18. Would you say that this child respects the property of others? |
| 19. Would you say that this child demonstrates self-control? |
| 20. Would you say that this child demonstrates respect for adults? |
| 21. Would you say that this child demonstrates respect for other children? |
| 22. Would you say that this child accepts responsibility for actions? |
| 23. Would you say that this child takes care of school materials? |
| 24. Would you say that this child shows tolerance to someone who made a mistake (e.g. when a child gives a wrong answer to a question posed by the teacher)? |
| **Sub.domain: Approaches to learning (8 items)** |
| 25. Would you say that this child listens attentively? |
| 26 . Would you say that this child completes work on time? |
| 27. Would you say that this child works independently? |
| 28 . Would you say that this child works neatly and carefully? |
| 29. Would you say that this child is able to solve day-to-day problems by him/herself? |
| 30. Would you say that this child is able to follow one-step instructions? |
| 31. Would you say that this child is able to follow class routines without reminders? |
| 32. Would you say that this child is able to adjust to changes in routines? |
| **Sub-domain: Readiness to explore new things (4 items)** |
| 33. Would you say that this child is curious about the world? |
| 34. Would you say that this child is eager to play with a new toy? |
| 35. Would you say that this child is eager to play a new game? |
| 36. Would you say that this child is eager to play with/read a new book? |
| **Sub-domain: Pro-social and helping behaviour (8 items)** |
| 37. Would you say that this child will try to help someone who has been hurt? |
| 38. Would you say that this child volunteers to help clear up a mess someone else has made? |
| 39. Would you say that this child, if there is a quarrel or dispute, will try to stop it? |
| 40. Would you say that this child offers to help other children who have difficulty with a task? |
| 41. Would you say that this child comforts a child who is crying or upset? |
| 42. Would you say that this child spontaneously helps to pick up objects which another child has dropped (e.g. pencils, books)? |
| 43. Would you say that this child will invite others to join in a game? |
| 44. Would you say that this child will help others who are feeling sick? |
| **Sub-domain: Anxious and fearful behaviour (5 items)** |
| 45. Would you say that this child seems to be unhappy, sad or depressed? |
| 46. Would you say that this child appears worried? |
| 47. Would you say that this child cries a lot? |
| 48. Would you say that this child is nervous, highly strung or tense? |
| 49. Would you say that this child is incapable of making decisions? |
| **Sub-domain: Aggressive behaviour (7 items)** |
| 50. Would you say that this child gets into physical fights? |
| 51. Would you say that this child bullies or is mean to others? |
| 52. Would you say that this child kicks, bites, hits other children or adults? |
| 53. Would you say that this child takes things that do not belong to him/her? |
| 54. Would you say that this child laughs at other children’s discomfort? |
| 55. Would you say that this child is disobedient? |
| 56. Would you say that this child has temper tantrums? |
| **Sub-domain: Hyperactive and inattentive behaviour (6 items)** |
| 57. Would you say that this child can’t sit still, is restless? |
| 58. Would you say that this child is distractible, has trouble sticking to any activity? |
| 59. Would you say that this child is impulsive, acts without thinking? |
| 60. Would you say that this child has difficulty awaiting turn in games or groups? |
| 61. Would you say that this child cannot settle to anything for more than a few moments? |
| 62. Would you say that this child is inattentive? |
| **Sub-domains: Interest in literacy/numeracy and memory (5 items)** |
| 71. Would you say this child is generally interested in books (pictures and print)? |
| 72. Would you say this child is interested in reading (inquisitive/curious about the meaning of printed material)? |
| 73. Would you say this child is able to remember things easily? |
| 74. Would you say this child is interested in mathematics? |
| 75. Would you say this child is interested in games involving numbers? |
| **Sub-domains: Basic literacy skills (8 items)** |
| 63. Would you say this child knows how to handle a book (e.g. turn a page)? |
| 64. Would you say this child is able to identify some letters of the alphabet? |
| 65. Would you say this child is able to attach sounds to letters? |
| 66. Would you say this child is showing awareness of rhyming words? |
| 67. Would you say this child is able to participate in group reading activities? |
| 68. Would you say this child is experimenting with writing tools? |
| 69. Would you say this child is aware of writing directions in English (left to right, top to bottom)? |
| 70. Would you say this child is able to write his/her own name in English? |
| **Sub-domains: Advanced literacy skills (6 items)** |
| 76. Would you say this child is able to read simple words? |
| 77. Would you say this child is able to read complex words? |
| 78. Would you say this child is able to read simple sentences? |
| 79. Would you say this child is interested in writing voluntarily (and not only under the teacher’s direction)? |
| 80. Would you say this child is able to write simple words? |
| 81. Would you say this child is able to write simple sentences? |
| **Sub-domains: Basic numeracy (7 items)** |
| 82. Would you say this child is able to sort and classify objects by common characteristics (e.g. shape, colour, size)? |
| 83. Would you say this child is able to use one.to.one correspondence? |
| 84. Would you say this child is able to count to twenty? |
| 85. Would you say this child is able to recognise numbers one to ten? |
| 86. Would you say this child is able to say which number is bigger of the two? |
| 87. Would you say this child is able to recognise geometric shapes (e.g. triangle, circle, square)? |
| 88. Would you say this child understands simple time concepts (e.g. today, summer, bedtime)? |

## S2 Table: Proportion of children (%) developmental vulnerable on each of the AEDC sub-domains in the self-regulation and executive function latent construct and early literacy/numeracy latent construct

| AEDC sub-domains | **Study cohort** | **No Year 3 NAPLAN** | **Non-participation in NAPLAN** | **No NTG preschool and /or no EY attendance records** |
| --- | --- | --- | --- | --- |
| **Number of Children, n** | 3,199 | 1,779 | 846 | 902 |
| **Proportion of children developmentally vulnerable (%)** | | | | |
| **Self-regulation and executive function** |  |  |  |  |
| Overall Social Competence | 9.3 | 7.5 | 19.7 | 8.6 |
| Responsibility and respect | 18.5 | 14.5 | 40.6 | 18.9 |
| Approaches to learning | 12.8 | 11.0 | 32.7 | 14.0 |
| Readiness to explore new things | 11.3 | 9.2 | 25.3 | 12.0 |
| Pro-social and helping behaviour | 13.2 | 10.4 | 29.5 | 11.7 |
| Anxious and fearful behaviour | 12.1 | 11.3 | 20.9 | 13.0 |
| Aggressive behaviour | 18.8 | 15.0 | 32.2 | 19.6 |
| Hyperactive and inattentive behaviour | 16.2 | 13.1 | 27.8 | 15.8 |
| Interest in literacy/numeracy and memory | 11.0 | 8.0 | 25.5 | 12.0 |
| **Early literacy/numeracy skills** |  |  |  |  |
| Basic literacy | 23.0 | 14.6 | 59.3 | 24.1 |
| Advanced literacy | 19.8 | 13.9 | 46.5 | 19.1 |
| Basic numeracy | 26.8 | 17.3 | 58.6 | 23.2 |

Note: The proportion of children identified as developmentally vulnerable in each of the AEDC sub-domains was calculated based on non-missing records. The proportion of children identified as developmentally vulnerable in each of the sub-domains were children who scored in the bottom 10% of the national AEDC population at age 5 years.

## S3 Table: Proportion of children (%) with missing data on each of the AEDC sub-domains in the self-regulation and executive function latent construct and early literacy/numeracy latent construct

| AEDC sub-domains | **Study cohort** | **No Year 3 NAPLAN** | **Non-participation in NAPLAN** | **No NTG preschool and /or no EY attendance records** |
| --- | --- | --- | --- | --- |
| **Number of Children, n** | 3,199 | 1,779 | 846 | 902 |
| **Proportion of children developmentally vulnerable (%)** | | | | |
| **Self-regulation and executive function** |  |  |  |  |
| Overall Social Competence | 3.1 | 4.3 | 17.4 | 3.3 |
| Responsibility and respect | 3.1 | 4.4 | 17.4 | 3.1 |
| Approaches to learning | 3.1 | 4.4 | 17.8 | 3.1 |
| Readiness to explore new things | 3.8 | 5.1 | 19.0 | 5.1 |
| Pro-social and helping behaviour | 6.3 | 6.6 | 21.7 | 6.0 |
| Anxious and fearful behaviour | 3.8 | 5.1 | 20.2 | 4.0 |
| Aggressive behaviour | 3.8 | 5.0 | 19.6 | 3.8 |
| Hyperactive and inattentive behaviour | 3.7 | 4.9 | 19.5 | 3.7 |
| Interest in literacy/numeracy and memory | 4.2 | 6.9 | 18.4 | 3.9 |
| **Early literacy/numeracy skills** |  |  |  |  |
| Basic literacy | 3.3 | 6.2 | 17.7 | 3.4 |
| Advanced literacy | 3.4 | 6.6 | 17.6 | 4.2 |
| Basic numeracy | 3.4 | 6.6 | 18.0 | 3.5 |

Note:

In the descriptive analysis (i.e. Table 1), the proportion of children identified as developmentally vulnerable in each of the sub-domains was presented and calculated based on non-missing records.

In the SEM, the standardised score from each of the sub-domains was used as manifest indicator variables for the two latent constructs ‘early literacy/numeracy skills’ and ‘self-regulation and executive function’.

In the conventional way to handle missing data, there were two major approaches with good statistical properties that produce unbiased results: maximum likelihood (ML) and multiple imputation (MI). In our analysis involving structural equation model, we have used the robust maximum likelihood with missing values (MLMV) estimator to handle missing data. The decision is based on the four arguments put forward by Allison (2012):

1. With MI, there is always a potential conflict between the imputation model and the analysis model. There is no potential conflict in ML because everything is done under one model.
2. The implementation of MI requires many different decisions, each of which involves uncertainty. ML involves far fewer decisions.
3. For a given set of data, ML always produces the same result. On the other hand, MI gives a different result every time you use it.
4. For a given set of data, ML always produces the same result. On the other hand, MI gives a different result every time you use it.

Reference: Allison PD. Handling missing data by maximum likelihood. In SAS global forum 2012 Apr 22 (Vol. 2012, No. 312, pp. 1038-21).

## S4 Table: Proportion of children (%) developmental vulnerable on each of the AEDC sub-domains, median school attendance rate (i.e. preschool and early year attendance) and proportion (%) of children at/above NMS by demographic variables, NT Aboriginal children

|  | **Sex** | |  | **ESB** | |  | **Remote** | |  | **SES** | |
| --- | --- | --- | --- | --- | --- | --- | --- | --- | --- | --- | --- |
|  | **Girl** | **Boy** |  | **NESB** | **ESB** |  | **No** | **Yes** |  | **Other** | **Lowest** |
| **Number of Children, n** | 728 | 704 |  | 1005 | 427 |  | 354 | 1078 |  | 701 | 731 |
| **Proportion of children developmentally vulnerable (%)** | | | | | | | | | | | |
| **Self-regulation and executive function** |  |  |  |  |  |  |  |  |  |  |  |
| Overall Social Competence | 10.7 | 17.2 |  | 14.2 | 13.1 |  | 15.0 | 13.5 |  | 14.5 | 13.2 |
| Responsibility and respect | 23.1 | 34.5 |  | 33.7 | 17.8 |  | 18.2 | 32.4 |  | 24.1 | 33.3 |
| Approaches to learning | 14.8 | 26.7 |  | 24.6 | 11.9 |  | 17.1 | 21.9 |  | 19.8 | 21.5 |
| Readiness to explore new things | 13.9 | 15.8 |  | 16.1 | 12.0 |  | 13.5 | 15.3 |  | 13.0 | 16.6 |
| Pro-social and helping behaviour | 15.6 | 24.3 |  | 23.8 | 11.8 |  | 13.3 | 22.2 |  | 15.1 | 24.6 |
| Anxious and fearful behaviour | 13.3 | 18.7 |  | 17.0 | 13.6 |  | 12.2 | 17.2 |  | 13.7 | 18.2 |
| Aggressive behaviour | 23.6 | 37.4 |  | 36.0 | 18.1 |  | 16.0 | 35.3 |  | 27.4 | 33.3 |
| Hyperactive and inattentive behaviour | 15.8 | 31.9 |  | 26.1 | 18.4 |  | 20.7 | 24.7 |  | 22.5 | 24.9 |
| Interest in literacy/numeracy and memory | 13.0 | 22.0 |  | 19.0 | 13.8 |  | 14.2 | 18.5 |  | 16.7 | 18.1 |
| **Early literacy/numeracy skills** | | | | | | | | | | | |
| Basic literacy | 37.5 | 45.1 |  | 50.8 | 20.2 |  | 20.6 | 48.4 |  | 32.5 | 49.9 |
| Advanced literacy | 26.3 | 39.8 |  | 39.6 | 18.3 |  | 20.3 | 37.3 |  | 29.0 | 36.8 |
| Basic numeracy | 43.5 | 51.0 |  | 56.3 | 27.4 |  | 27.0 | 54.3 |  | 38.3 | 56.0 |
| **Median of school attendance (out of 100)** | | | | | | | | | | | |
| Preschool attendance | 64.0 | 67.4 |  | 58.2 | 83.1 |  | 82.3 | 60.6 |  | 73.5 | 58.8 |
| Early year attendance | 77.3 | 77.4 |  | 70.6 | 88.8 |  | 88.5 | 72.7 |  | 84.2 | 71.4 |
| **Proportion of children at/above NMS (%)** | | | | | | | | | | | |
| Year 3 NAPLAN Reading | 50.5 | 46.0 |  | 36.8 | 75.4 |  | 74.6 | 39.7 |  | 58.3 | 38.7 |
| Year 3 NAPLAN Numeracy | 56.7 | 47.0 |  | 37.3 | 86.4 |  | 85.3 | 41.0 |  | 65.0 | 39.4 |

Note: The proportion of children identified as developmentally vulnerable in each of the AEDC sub-domains was calculated based on non-missing records.

The proportion of children identified as developmentally vulnerable in each of the sub-domains were children who scored in the bottom 10% of the national AEDC population at age 5 years.

Under the NAPLAN assessment scale, there are 10 bands and the second lowest band reported for each year level represents the national minimum standard (NMS) expected of students at that year level; NMS represents the benchmark for the basic level of knowledge and understanding that a student requires to function at the specific year level in Australia.

The socio-economic status (SES) was measured with the Index of Relative Socio-Economic Disadvantage (IRSD) representing socio-economic disadvantage; in the descriptive analysis, there were two categories: the most socio-economically disadvantaged regions (the most disadvantaged deciles of IRSD) and other less socio-economically disadvantaged regions (the other nine deciles).

## S5 Table: Proportion of children (%) developmental vulnerable on each of the AEDC sub-domains, median school attendance rate (i.e. preschool and early year attendance) and proportion (%) of children at/above NMS by demographic variables, NT non-Aboriginal children

|  | **Sex** | |  | **ESB** | |  | **Remote** | | |  | **SES** | | |  |
| --- | --- | --- | --- | --- | --- | --- | --- | --- | --- | --- | --- | --- | --- | --- |
|  | **Girl** | **Boy** |  | **NESB** | **ESB** |  | **No** | **Yes** |  | **Other** | | **Lowest** | | |
| **Number of Children, n** | 887 | 880 |  | 299 | 1468 |  | 1343 | 424 | |  | 1553 | | 213 |  |
| **Proportion of children developmentally vulnerable (%)** | | | | | | | | | | | | | |  |
| **Self-regulation and executive function** |  |  |  |  |  |  |  |  | |  |  | |  |  |
| Overall Social Competence | 3.6 | 8.0 |  | 9.5 | 5.1 |  | 6.4 | 3.8 | |  | 5.8 | | 5.7 |  |
| Responsibility and respect | 7.9 | 13.7 |  | 12.8 | 10.4 |  | 11.3 | 9.3 | |  | 10.9 | | 9.5 |  |
| Approaches to learning | 4.2 | 9.6 |  | 12.8 | 5.7 |  | 7.4 | 5.2 | |  | 7.2 | | 4.3 |  |
| Readiness to explore new things | 7.8 | 9.3 |  | 15.1 | 7.3 |  | 9.4 | 6.0 | |  | 9.0 | | 5.7 |  |
| Pro-social and helping behaviour | 4.2 | 12.6 |  | 15.4 | 6.9 |  | 8.5 | 7.9 | |  | 8.2 | | 9.0 |  |
| Anxious and fearful behaviour | 7.7 | 10.8 |  | 9.8 | 9.1 |  | 9.6 | 8.2 | |  | 9.2 | | 9.6 |  |
| Aggressive behaviour | 6.6 | 13.7 |  | 9.5 | 10.2 |  | 10.2 | 9.6 | |  | 9.6 | | 13.5 |  |
| Hyperactive and inattentive behaviour | 5.3 | 15.9 |  | 12.9 | 10.1 |  | 11.6 | 7.2 | |  | 10.8 | | 8.2 |  |
| Interest in literacy/numeracy and memory | 4.4 | 8.0 |  | 10.3 | 5.4 |  | 6.6 | 5.0 | |  | 6.3 | | 5.2 |  |
| **Early literacy/numeracy skills** | | | | | | | | | | | | | |  |
| Basic literacy | 5.7 | 12.7 |  | 16.3 | 7.7 |  | 8.7 | 10.7 | |  | 9.5 | | 7.1 |  |
| Advanced literacy | 5.2 | 14.7 |  | 16.9 | 8.5 |  | 9.7 | 10.5 | |  | 10.1 | | 8.5 |  |
| Basic numeracy | 9.4 | 13.2 |  | 19.3 | 9.7 |  | 10.3 | 14.3 | |  | 11.4 | | 10.4 |  |
| **Median of school attendance (out of 100)** | | | | | | | | | | | | | |  |
| Preschool attendance | 91.0 | 90.9 |  | 89.2 | 91.3 |  | 91.1 | 90.0 | |  | 91.1 | | 89.2 |  |
| Early year attendance | 93.4 | 93.5 |  | 91.9 | 93.6 |  | 93.5 | 93.0 | |  | 93.5 | | 92.9 |  |
| **Proportion of children at/above NMS (%)** | | | | | | | | | | | | | |  |
| Year 3 NAPLAN Reading | 93.6 | 88.8 |  | 90.0 | 91.4 |  | 91.4 | 90.6 | |  | 91.1 | | 91.5 |  |
| Year 3 NAPLAN Numeracy | 98.0 | 93.6 |  | 95.0 | 96.0 |  | 96.4 | 94.1 | |  | 95.8 | | 95.8 |  |

Note: The proportion of children identified as developmentally vulnerable in each of the AEDC sub-domains was calculated based on non-missing records.

The proportion of children identified as developmentally vulnerable in each of the sub-domains were children who scored in the bottom 10% of the national AEDC population at age 5 years.

Under the NAPLAN assessment scale, there are 10 bands and the second lowest band reported for each year level represents the national minimum standard (NMS) expected of students at that year level; NMS represents the benchmark for the basic level of knowledge and understanding that a student requires to function at the specific year level in Australia.

The socio-economic status (SES) was measured with the Index of Relative Socio-Economic Disadvantage (IRSD) representing socio-economic disadvantage; in the descriptive analysis, there were two categories: the most socio-economically disadvantaged regions (the most disadvantaged deciles of IRSD) and other less socio-economically disadvantaged regions (the other nine deciles).

## S6 Table. Correlation table for co-variates

| **Aboriginal children** | | | | | | | | | |
| --- | --- | --- | --- | --- | --- | --- | --- | --- | --- |
|  | **1** | **2** | **3** | **4** | **5** | **6** | **7** | **8** | **9** |
| **1.Male** | 1 |  |  |  |  |  |  |  |  |
| **2.Non-English speaking background** | 0 | 1 |  |  |  |  |  |  |  |
| **3.Remote** | -0.04 | 0.64* | 1 |  |  |  |  |  |  |
| **4.Socio-economic status (decile)** | 0.02 | -0.37* | -0.47* | 1 |  |  |  |  |  |
| **5.Self-regulation and executive function** | -0.23* | -0.20* | -0.16* | 0.13* | 1 |  |  |  |  |
| **6.Preschool attendance** | 0.02 | -0.40* | -0.28* | 0.24* | 0.15* | 1 |  |  |  |
| **7.Early literacy/numeracy skills** | -0.11* | -0.34* | -0.28* | 0.18* | 0.57* | 0.37* | 1 |  |  |
| **8.Early years attendance** | 0 | -0.47* | -0.39* | 0.28* | 0.21* | 0.65* | 0.40* | 1 |  |
| **9.Year 3 reading/numeracy** | 0 | -0.49* | -0.43* | 0.34* | 0.27* | 0.35* | 0.43* | 0.41* | 1 |
| **Non-Aboriginal children** | | | | | | | | | |
|  | **1** | **2** | **3** | **4** | **5** | **6** | **7** | **8** | **9** |
| **1.Male** | 1 |  |  |  |  |  |  |  |  |
| **2.Non-English speaking background** | 0.02 | 1 |  |  |  |  |  |  |  |
| **3.Remote** | 0.03 | -0.15* | 1 |  |  |  |  |  |  |
| **4.Socio-economic status(decile)** | 0.01 | 0.03 | -0.45* | 1 |  |  |  |  |  |
| **5.Self-regulation and executive function** | -0.19* | -0.09* | 0.04 | 0.06 | 1 |  |  |  |  |
| **6.Preschool attendance** | -0.05 | -0.11* | -0.02 | 0.11* | 0.15* | 1 |  |  |  |
| **7.Early literacy/numeracy skills** | -0.14* | -0.16* | -0.02 | 0.02 | 0.51* | 0.17* | 1 |  |  |
| **8.Early years attendance** | -0.03 | -0.09* | -0.05 | 0.08 | 0.10* | 0.37* | 0.13* | 1 |  |
| **9.Year 3 reading/numeracy** | -0.02 | -0.08 | -0.02 | 0.12* | 0.28* | 0.11* | 0.43* | 0.11* | 1 |

## S7 Table: Standardised direct and indirect effects (i.e. β) of self-regulation/executive function and preschool attendance on Year 3 academic outcomes with early literacy/numeracy skills and early years attendance as mediators for both Aboriginal and non-Aboriginal children by sex, English speaking background (ESB), remoteness and socio-economic status (SES), respectively

| **Pathways to NAPLAN in mediation model** | **Sex** | | |  | **ESB?** | | |  | **Remoteness** | | |  | **SES** | | |
| --- | --- | --- | --- | --- | --- | --- | --- | --- | --- | --- | --- | --- | --- | --- | --- |
|  | **Girl** | **Boy** | **p** |  | **ESB** | **NSEB** | **p** |  | **Urban** | **Remote** | **p** |  | **Other** | **Lowest** | **p** |
| **Aboriginal children** |  |  |  |  |  |  |  |  |  |  |  |  |  |  |  |
| **Direct effects** |  |  |  |  |  |  |  |  |  |  |  |  |  |  |  |
| **Early literacy and numeracy skills** | 0.13 | 0.35 | 0.048 |  | 0.61 | 0.09 | 0.000 |  | 0.63 | 0.14 | 0.001 |  | 0.43 | 0.24 | 0.001 |
| **Early years attendance** | 0.34 | 0.26 | 0.321 |  | 0.14 | 0.37 | 0.054 |  | 0.17 | 0.36 | 0.158 |  | 0.09 | 0.16 | 0.785 |
| **Indirect effects** |  |  |  |  |  |  |  |  |  |  |  |  |  |  |  |
| **Executive functioning and self-regulation** | 0.12 | 0.28 | 0.059 |  | 0.47 | 0.11 | 0.000 |  | 0.48 | 0.14 | 0.004 |  | 0.31 | 0.20 | 0.004 |
| **Preschool attendance** | 0.21 | 0.21 | 0.338 |  | 0.23 | 0.23 | 0.554 |  | 0.25 | 0.23 | 0.754 |  | 0.14 | 0.14 | 0.030 |
| **non-Aboriginal children** |  |  |  |  |  |  |  |  |  |  |  |  |  |  |  |
| **Direct effects** |  |  |  |  |  |  |  |  |  |  |  |  |  |  |  |
| **Early literacy and numeracy skills** | 0.38 | 0.67 | 0.019 |  | 0.51 | 0.71 | 0.785 |  | 0.59 | 0.28 | 0.002 |  | 0.51 | 0.59 | 0.800 |
| **Early years attendance** | 0.02 | 0.07 | 0.150 |  | 0.05 | 0.09 | 0.833 |  | 0.05 | 0.11 | 0.297 |  | 0.04 | 0.07 | 0.808 |
| **Indirect effects** |  |  |  |  |  |  |  |  |  |  |  |  |  |  |  |
| **Executive functioning and self-regulation** | 0.25 | 0.49 | 0.004 |  | 0.35 | 0.54 | 0.595 |  | 0.41 | 0.22 | 0.089 |  | 0.36 | 0.48 | 0.443 |
| **Preschool attendance** | 0.03 | 0.05 | 0.115 |  | 0.05 | 0.04 | 0.398 |  | 0.04 | 0.05 | 0.945 |  | 0.04 | 0.05 | 0.850 |

Note: The socio-economic status (SES) was measured with the Index of Relative Socio-Economic Disadvantage (IRSD) representing socio-economic disadvantage. In the stratified analysis, there were two categories: the most socio-economically disadvantaged regions (the most disadvantaged deciles of IRSD) and other less socio-economically disadvantaged regions (the other nine deciles).
